# Geopolymer-Assisted Conversion of Cs-Exchanged 13X Zeolite into Stable Cs-Aluminosilicates for Long-Term Cesium Immobilization

**DOI:** 10.3390/ma19153176

**Published:** 2026-07-24

**Authors:** Mia Omerašević, Sema Erentürk, Nataša Mladenović Nikolić, Miomir Krsmanović, Nada Adamović, Ljiljana Kljajević, Dušan Bučevac

**Affiliations:** 1 Vinča Institute of Nuclear Sciences, National Institute of the Republic of Serbia, University of Belgrade, 11351 Belgrade, Serbia; mia@vin.bg.ac.rs (M.O.); natasa.nikolic@vin.bg.ac.rs (N.M.N.); miomir.krsmanovic@vin.bg.ac.rs (M.K.); nada.adamovic@vin.bg.ac.rs (N.A.); ljiljana@vin.bg.ac.rs (L.K.); 2 Energy Institute, Istanbul Technical University, 34469 Istanbul, Turkey; erenturk@itu.edu.tr

**Keywords:** Cs disposal, ion exchange, zeolite, geopolymer, heat treatment

## Abstract

**Highlights:**

- Cs^+^ ions were removed from water by ion exchange using Na-13X zeolite, which was subsequently incorporated into geopolymer matrix.
- Heat treatment of the geopolymer/Cs-13X zeolite composites at 950 °C induced crystallization of two stable Cs-bearing phases.
- Heat treatment promoted the partitioning of Cs between the stable crystalline phases originating from the zeolite and geopolymer matrix.
- The highest values of crystallinity, bulk density, and compressive strength were measured for samples containing 50 wt% Cs-13X zeolite.

**Abstract:**

A promising approach for the removal of Cs^+^ ions from aqueous solutions and their long-term immobilization was investigated, as the safe containment of radioactive cesium is essential due to its long half-life and environmental hazards. The approach involves ion exchange using zeolite, geopolymerization, and subsequent heat treatment of the geopolymer/zeolite composite. Cs^+^ ions were removed from water by ion exchange using Na-13X zeolite, in which Cs^+^ ions replaced Na^+^ ions within the zeolite framework. The resulting Cs-13X zeolite was subsequently combined with kaolinite to prepare geopolymer/Cs-13X zeolite composites containing 10–50 wt% Cs-13X zeolite. During geopolymerization, a portion of the Cs was released from Cs-13X zeolite and incorporated into the geopolymer matrix, while the remaining Cs remained immobilized within the zeolite structure. Heat treatment of the geopolymer/Cs-13X zeolite composites at 950 °C induced crystallization of two stable Cs-bearing phases. The Cs incorporated into the geopolymer matrix crystallized as the CAS phase, whereas the Cs retained in the Cs-13X zeolite crystallized as pollucite. The crystalline phase content in the heat-treated composite samples increased with Cs-13X zeolite loading, resulting in improved density and compressive strength. The highest values of crystallinity (72%), bulk density (2.42 g/cm^3^), and compressive strength (~53 MPa) were measured for samples containing 50 wt% Cs-13X zeolite. This level of compressive strength is sufficient to ensure safe handling and long-term containment of the immobilized Cs^+^ ions.

## 1. Introduction

Previous nuclear accidents have consistently highlighted the critical importance of the safe disposal of nuclear wastewater for protecting human health and supporting sustainable development. Nuclear wastewater exhibits severe radioactive hazards since it contains fission products with high decay heat and long half-life periods, such as Cs ^137^ (0.512 MeV, t_1/2_ = 30.17 years) [1,2]. To minimize radiological hazards, radionuclides such as cesium must be immobilized within a durable matrix and subsequently isolated through geological disposal. One of the most widely used approaches for treating and disposing of radioactive waste involves the use of ion-exchanged zeolites [3,4,5].

Zeolites are among the most extensively studied and cost-effective sorbent materials for the removal of radioactive contaminants from aqueous systems. They are more suitable than some other sorbents, such as organic ion-exchange resins and cementitious materials. The use of organic ion-exchange resins has been found to be unsuitable due to their low radiation and thermal stability, while cementitious materials have several disadvantages, including high porosity, poor adsorption capacity, and high cesium diffusivity, particularly in hydrated cement [6]. Zeolites, whether natural or synthetic, are aluminosilicate materials with a three-dimensional cage-like structure. They exhibit strong resistance to radiation damage [5] and a high affinity for cesium (Cs^+^) cations [3,7,8]. Sorption performance is primarily governed by their framework composition, particularly the Si/Al ratio, pore topology, and the type of exchangeable cations. Low-Si/Al zeolites, including clinoptilolite, chabazite, LTA, and FAU, generally exhibit the highest uptake of radioactive cations due to their higher framework charge and greater density of ion-exchange sites [9,10]. In addition to their high sorption efficiency, zeolites are considered low-cost materials because they are widely available as natural minerals, can be synthesized on a large scale, and can be produced from industrial by-products, such as coal fly ash, making them attractive sorbents for economically viable radioactive wastewater treatment [11,12,13].

After ion exchange and adsorption of Cs, it is crucial to immobilize the ions within the zeolite framework to prevent their release when the material comes into contact with water. Several strategies have been developed to minimize leaching. For example, the pores of zeolites can be sealed using barium to further restrict Cs ions’ mobility [14,15]. Additionally, Cs-loaded zeolites can be incorporated into borosilicate glass matrices [16]. In addition to the glass, cement-based materials are also widely proposed and investigated for use in the immobilization of low- and intermediate-level radioactive wastes [17,18]. Cementation of zeolite after ion exchange was the accepted route for intermediate-level waste in the UK [19]. However, hardened cement is a porous material with limited corrosion resistance and poor thermal stability [18]. As a result, cement is not considered as an optimal material for nuclear waste immobilization.

In recent years, geopolymers, which consist of interconnected Si–O–Al frameworks, have been increasingly recognized as an alternative to conventional cementitious materials. These materials, commonly referred to as inorganic polymeric binders or alkali-activated materials (AAMs), have been proposed as sustainable alternatives to conventional cement due to their favorable mechanical properties and reduced carbon footprint [20,21,22,23]. AAMs consist of aluminate and silicate groups that exhibit tetrahedral coordination. Aluminum, when bonded to four oxygen atoms, acquires a negative charge that is balanced by alkali cations, typically sodium (Na^+^) or potassium (K^+^). Therefore, the synthesis of geopolymers requires the presence of both aluminate and silicate precursors. Aluminosilicate materials are generally in a solid form, whereas additional silicate is commonly introduced as an alkaline silicate solution. Typical aluminosilicate sources include metakaolin (MK), coal fly ash, and metallurgical slags [18,22,24,25], which are activated using highly alkaline solutions or alkali silicate solutions [26,27]. Upon mixing, dissolution and precipitation processes occur, resulting in the formation of a three-dimensional geopolymer network structure [23]. Therefore, geopolymers have been suggested as an alternative matrix for encapsulating loaded zeolites [28].

Radionuclides can be more effectively immobilized in geopolymer–zeolite composites due to the dual protective effect provided by these two components [29]. However, the chemical stability of such composites remains a challenge, since they contain a significant amount of geopolymer gels that are susceptible to degradation in highly alkaline, acidic, and saline environments. Consequently, the release of radionuclides and a reduction in compressive strength may occur [30,31]. In addition, free and bound water present in geopolymer–zeolite composites can decompose under gamma irradiation, leading to the formation of hydrogen (H_2_), which increases the risk of hydrogen explosion [32,33,34]. Therefore, it is necessary to develop modified approaches that would improve the chemical stability and reduce the water content of geopolymer–zeolite composites. Both the free and bound water present in geopolymers and zeolites can evaporate during thermal treatment at elevated temperatures, thereby reducing the risk of hydrogen generation caused by water radiolysis [35]. Therefore, the transformation of geopolymer–zeolite composites into aluminosilicate ceramic forms may represent a sustainable approach for overcoming the aforementioned limitations. In addition, the available literature indicates that geopolymers and zeolites, as precursors for aluminosilicate ceramics, can be synthesized at relatively low temperatures, while the shape of the final waste forms can be readily controlled during the casting stage, facilitating their transportation and storage [36,37]. Consequently, an aluminosilicate ceramic matrix obtained by thermal treatment of metakaolin-based geopolymer–zeolite 13X composites may represent a promising option for radionuclide immobilization, and therefore its immobilization performance should be further investigated in detail.

Although numerous studies have investigated Cs^+^ removal by zeolites and the immobilization of Cs-containing wastes, the stabilization of Cs-loaded sorbents after the adsorption process has received considerably less attention. Moreover, the mechanisms governing Cs partitioning between zeolite and the geopolymer matrix during geopolymerization, as well as the phase evolution after heat treatment, remain insufficiently understood. Therefore, the aim of this study is to develop a thermal treatment that promotes the formation of stable Cs-containing phases at the lowest possible temperature in order to minimize Cs volatilization. The aim of this research was to investigate the combined approach of Cs removal using Na-13X zeolite followed by the immobilization of Cs-loaded zeolite in a geopolymer matrix. Particular attention was devoted to achieving the crystallization of stable Cs-bearing phases at a relatively low temperature of 950 °C in order to minimize Cs volatilization during thermal treatment. Furthermore, the study aimed to elucidate the distribution of Cs between the stable crystalline phases derived from the zeolite and geopolymer matrix. The detailed phase analysis provides new insights into the immobilization mechanisms and the long-term stabilization of spent Cs-loaded sorbents.

## 2. Materials and Methods

### 2.1. Materials

**Figure 1:**
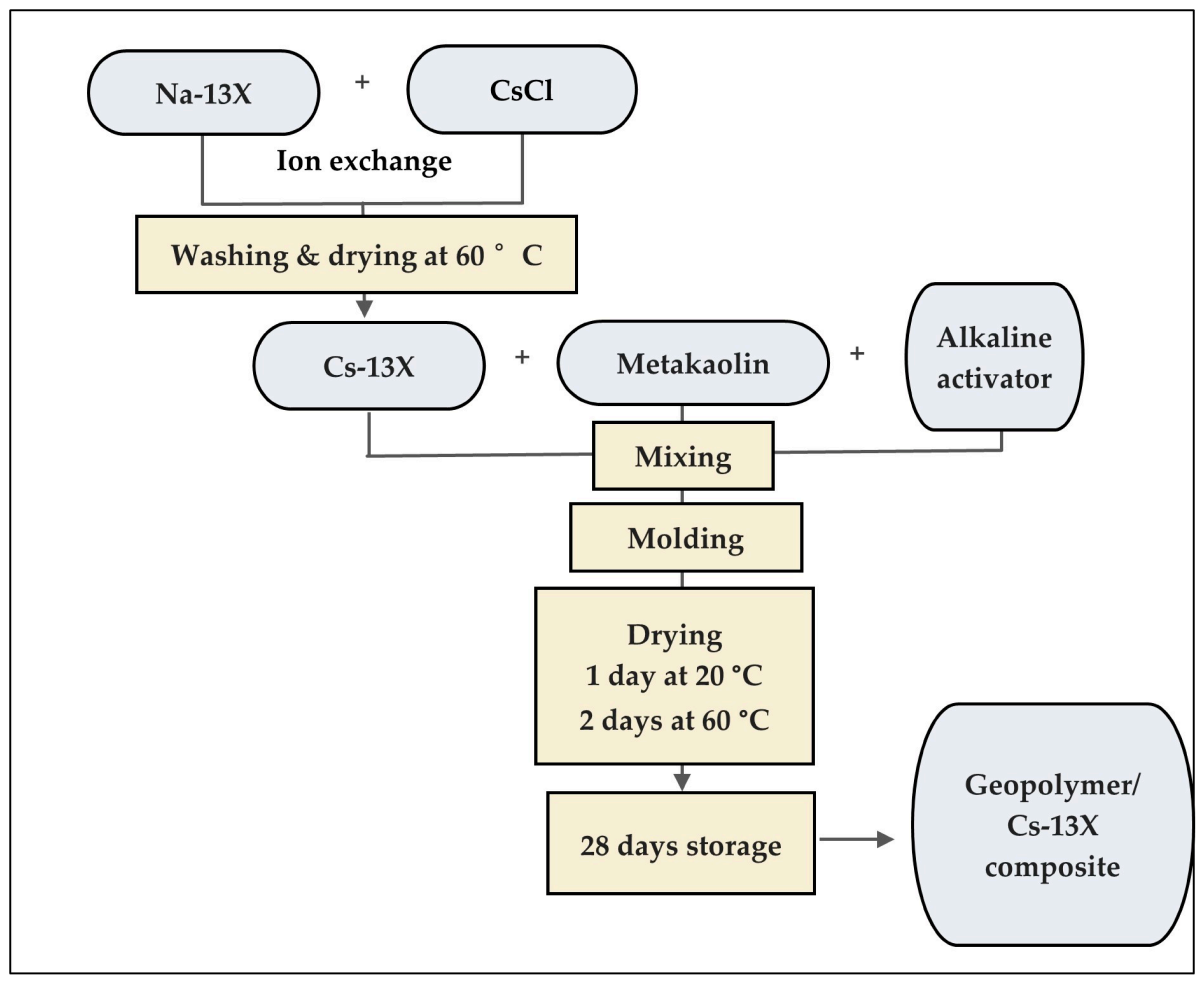
Schematic representation of the fabrication process of the geopolymer/Cs-13X zeolite composite.

The sodium form of X (FAU)-type (Si/Al = 1.26) zeolite (Molecular sieves 13X, powder Thermo Fisher-Scientific, Waltham, MA, USA) was used as a starting material and denoted as Na-13X. The material is characterized by a 12-membered ring pore opening of about 7.4 Å, leading to supercages of approximately 13 Å in diameter [38]. The specific surface area is ~6.25 m^2^/g, with a micropore volume of approximately 0.25–0.35 cm^3^ g^−1^ [13]. The partially Cs-exchanged form of Na-13X zeolite was prepared through three consecutive cation exchange cycles using a 0.1 M aqueous CsCl solution (Cesium Chloride p.a., Centrohem, Stara Pazova, Serbia, ≥99% CsCl), with a solid-to-liquid ratio of 1:20 (Figure 1). It is important to note that complete replacement of Na^+^ ions with Cs^+^ ions cannot be achieved under atmospheric pressure. However, it was found that three ion-exchange cycles represent the optimal number of cycles required to achieve a high degree of ion substitution. Each ion-exchange cycle was performed by stirring the zeolite suspension for 5 h at approximately 60 °C, after which the process was repeated twice under the same conditions. The degree of Na^+^ exchange was determined from the difference between the Na^+^ concentration in the zeolite before and after the ion exchange. Following the final exchange cycle, the Cs-exchanged 13X zeolite (hereafter denoted as Cs-13X) was thoroughly washed with distilled water to remove residual Cl^−^ ions and dried at 60 °C. Under these conditions, an almost complete exchange of Na^+^ with Cs^+^ was achieved, and the resulting Cs-13X zeolite exhibited an average particle size of approximately 2 μm.

Kaolin clay, sourced from Rudovci, Serbia, served as the raw material for the synthesis of alkali-activated composite materials (AACMs). Metakaolin (MK) was obtained through the calcination of kaolin at 750 °C for 3 h, which induced dehydroxylation of the clay mineral. The particle size analysis indicated a broad particle size distribution of the obtained metakaolin (MK). The characteristic particle diameters were d(0.1) = 4.05 µm, d(0.5) = 122.12 µm, and d(0.9) = 383.89 µm. The specific surface area (BET) was 16 m^2^/g.

The alkali-activating solution was prepared by combining sodium silicate (Na_2_SiO_3_, Galenika Magmasil, Belgrade, Serbia; molar modulus 3.10–3.40, SiO_2_, 25.20–28.00 wt%, Na_2_O 8.00–9.00 wt%) with sodium hydroxide (8 M, NaOH, Centrohem, Stara Pazova, Serbia). The chemical composition of the resulting metakaolin is summarized in Table 1.

### 2.2. Synthesis of Alkali-Activated Composite Materials Based on Metakaolin and Cs-13X Zeolite

Alkali-activated composite materials (AACMs) based on metakaolin and 10–50 wt% Cs-13X zeolite were synthesized through the dissolution of solid-phase precursor using an alkaline activator comprising sodium silicate (Na_2_SiO_3_) and 8 M sodium hydroxide (NaOH). The sodium silicate molar modulus (SiO_2_/Na_2_O) was 3.1. The volumetric ratio of NaOH to Na_2_SiO_3_ was maintained at 1:1.5. The alkali activator solution was prepared prior to synthesis and stirred on a magnetic stirrer for approximately three hours. The solid-phase precursors, metakaolin (MK) and Cs-13X zeolite, were homogenized before mixing with the alkaline activator to ensure compositional uniformity. The composition of the solid-phase precursor is presented in Table 2. The mass ratio of solid-phase precursors (MK/Cs-13X) to alkali activator solution was approximately 0.9–1.0 for all systems. The all-solid materials and activator solutions were thoroughly mixed, poured into 20 mm diameter molds, and left to set for one day at room temperature (20 ± 1 °C). Subsequently, the samples were cured in an oven at 60 °C for 2 days to promote the geopolymerization reaction [39]. The cured samples were then stored at room temperature for at least 28 days to allow aging and further structural development. After the aging period, the specimens were prepared for subsequent characterization and analysis. A scheme of the entire synthesis process is shown in Figure 1.

Composites, geopolymers with 10, 30, and 50% of Cs-13X zeolite, were heat-treated at 950 °C for 2 h in air. This temperature was selected as the highest possible sintering temperature to obtain a sample that is as dense as possible while avoiding potential Cs evaporation. Heating rate was 5 °C/min to prevent potential cracking of the samples during water evaporation.

### 2.3. Methods

X-ray Fluorescence (XRF) analysis of samples before and after ion exchange was performed using a Thermo Scientific ARL Perform’X Sequential X-ray Fluorescence Spectrometer (Waltham, MA, USA), equipped with a Rh target tube, seven monochromators, and a wavelength-dispersive spectrometer. A vacuum was used as the medium for analyses to avoid the interaction of X-rays with air particles. The data were acquired and processed using Thermo Scientific UniQuant 3.0 Analysis Software. The phase analysis of zeolite, geopolymers and geopolymer/zeolite composites was done by means of X-ray diffraction (XRD) using a Rigaku Ultima IV (Rigaku Corporation, Tokyo, Japan) diffractometer with filtered Cu Kα radiation. The diffraction data of samples were collected in the 2*θ* range, from 4° to 70° using a step size of 0.02°, for routine phase analysis. The present phases were identified with the help of the PDXL2 software (version 2.0.3.0), with reference to the patterns of the International Center for Diffraction Data (ICDD), version 2023. The amorphous/crystalline phase ratio in samples heat-treated at 950 °C was obtained by the integration method with the help of the Origin program (2026, 21-day free trial). This procedure utilizes a straight line as a baseline, established using the highest 2*θ* values to approximate the continuous background signal, and compares the area beneath the entire diffraction pattern with that beneath the crystalline peaks, according to the following equation [40]:

(1) $$Crystallinity\;\left(\%\right)=100\;\times \;\frac{Area\;crystalline\;XRD\;peaks}{Area\;under\;all\;XRD\;peaks}$$

Bulk density and open porosity were determined by the Archimedes’ method according to the ASTM C373-18(2023) Standard Test Method [41]. Five samples of each composition were tested, and the average value was calculated.

The bulk density (*B*) was measured using the following equation:

(2) $$B\;\left(\frac{g}{{cm}^{3}}\right)=\;\frac{D}{M-S}$$

where *D* is the mass of the dry sample, *M* is the saturated mass (after soaking the sample in water), and *S* is the mass of the sample suspended in water.

The open porosity (*OP*) was measured using the following equation:

(3) $$OP\;\left(\%\right)=\;\frac{M-D}{M-S}\times 100$$

The difference in *M* − *D* represents the volume of open pores, i.e., volume of water that entered the open pores, whereas *M* − *S* represents the total volume of the specimen.

Morphological analysis of all samples before and after heat treatment was performed using a JEOL JSM-6610LV (JEOL Ltd., Tokyo, Japan) scanning electron microscope (SEM). The samples were coated with a layer of gold, and the secondary electron images were acquired using 0.1 nA beam of electrons accelerated at 20 and 30 kV. Room-temperature compressive tests of cylindrical samples were carried out on an Instron Universal Testing Machine (Model 1185, Norwood, MA, USA) with a 500 kg load cell according to the ASTM C1424-15(2019) Standard Test Method for Monotonic Compressive Strength of Advanced Ceramics at Ambient Temperature [42]. The average value of a minimum of six samples was calculated. The upper and bottom surfaces of the cylindrical samples were carefully ground to make them parallel.

## 3. Results and Discussion

### 3.1. Characterization of Starting Materials

The chemical composition of Na–13X zeolite before and after Cs^+^ ion exchange is presented in Table 3. During the exchange process, Cs^+^ ions migrate through the pores and channels of the zeolite structure and replace the exchangeable original Na^+^ cations. The resulting cesium-exchanged zeolite contains approximately 21 wt% of Cs^+^ ions. The obtained data confirm efficient substitution of Na^+^ by Cs^+^, high cesium incorporation in the zeolite structure, and a preservation of the aluminosilicate framework, i.e., the Si/Al ratio before and after ion exchange. It is important to note that the Si/Al ratio remains typical for 13X zeolite (1.2–1.5). The calculated Cs^+^ → Na^+^ ion-exchange degree was 62%, indicating that approximately two-thirds of the cation-exchange sites were occupied by Cs^+^ ions, confirming the successful ion-exchange process.

**Figure 2:**
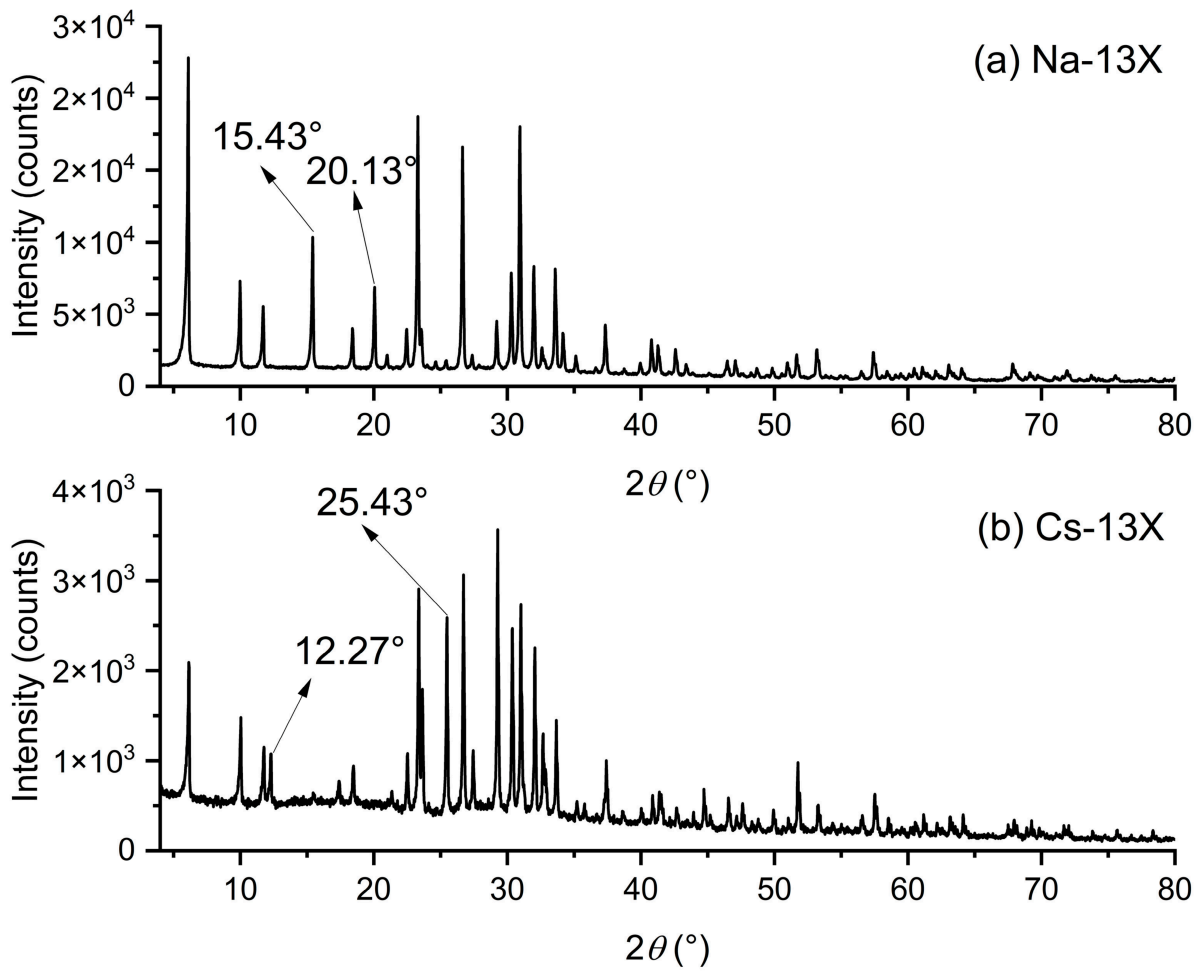
XRD patterns of (**a**) Na-13X (PDF 00-038-0237) and (**b**) Cs-13X (PDF 01-076-6877).

**Figure 3:**
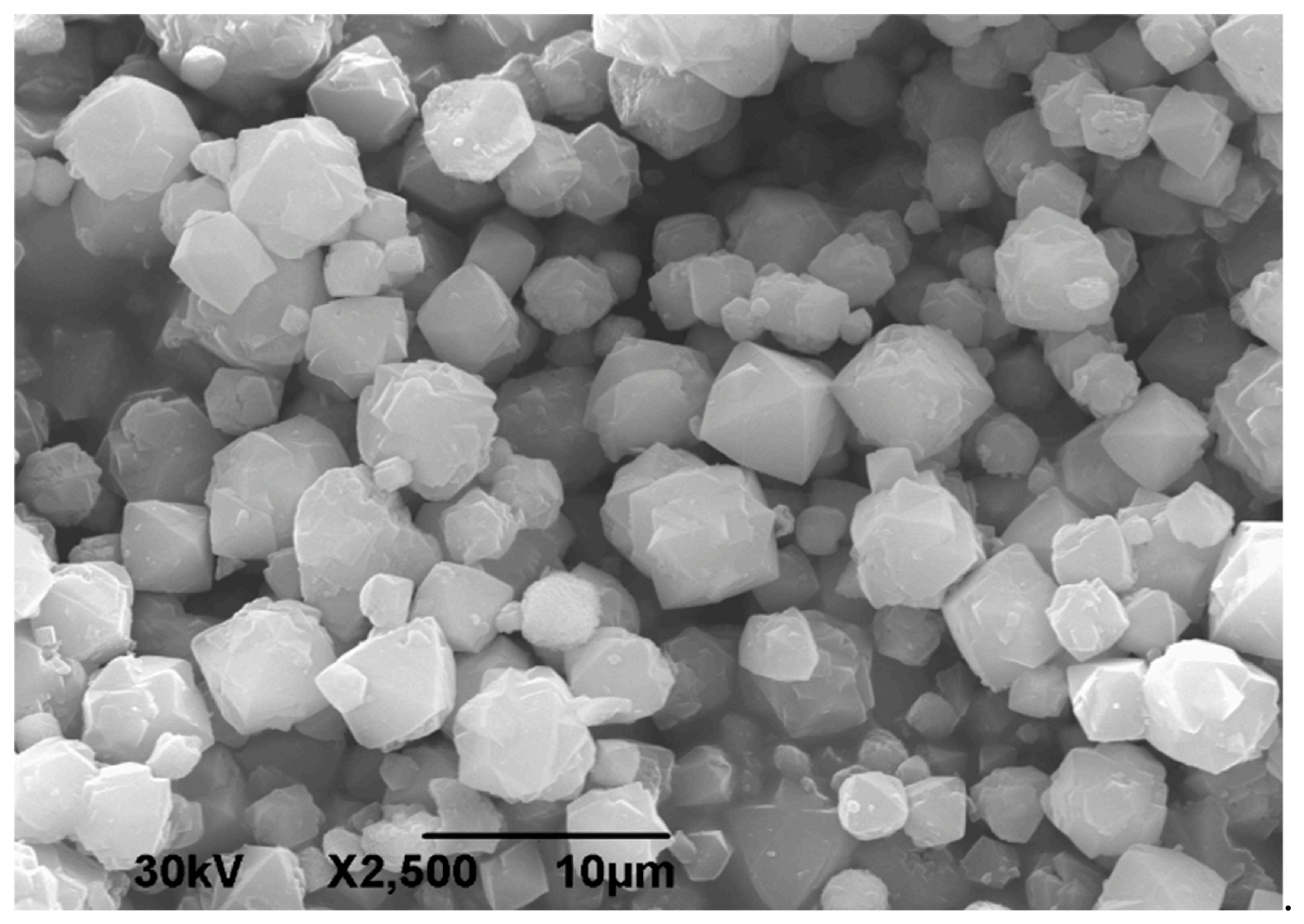
SEM micrograph of Cs-13X zeolite powder.

X-ray diffractograms of Na-13X zeolite before and after ion exchange are presented in Figure 2. As mentioned, ion exchange of Na^+^ with Cs^+^ in the Na-13X zeolite framework results in the formation of Cs-13X zeolite. As Figure 2 indicates, the degree of crystallinity of newly formed Cs-13X zeolite is significantly lower than that of Na-13X. To be more precise, the amount of amorphous phase increased after ion exchange. This is expected given that the ionic radius of Cs^+^ (167 pm) is considerable larger than that of Na^+^ (102 pm) [43], causing a certain degree of distortion in the crystal structure upon substitution of Na^+^ by Cs^+^. Furthermore, the intensity of the reflections is considerably lower than that in the initial zeolite sample. These changes in reflection intensity during ion exchange were also observed by Gu et al. [16], who attributed them to changes in X-ray structural factors caused by the introduction of Cs^+^ into the crystal structure. Figure 2 also shows that two XRD patterns are very similar indicating that the alumosilicate framework remains mainly intact after ion exchange and that the substitution of Na^+^ by Cs^+^ does not significantly alter the crystal structure. It can be observed that two reflections at 2*θ* = 15.43° and 20.13°, which belong to the Na-13X zeolite, are absent in the diffractogram of the modified zeolite, while new reflections appear at 2*θ* = 25.43° and 12.27°. The appearance of new reflections was explained by Gu et al. as the formation of a secondary phase, which is actually a Cs-rich aluminosilicate [16]. Cesium, as a cation with a greater number of electrons than Na, scatters X-rays much more effectively, causing an increase in intensity of reflections related to those planes in which Cs^+^ cations are located. As Figure 3 evidences, the Cs-13X zeolite powder comprises agglomerates and well-defined, multifaceted rhombohedral particles with sharp edges, ranging in size from 1 to 7 µm.

### 3.2. Characterization of Geopolymer/Cs-13X Zeolite Composite

**Figure 4:**
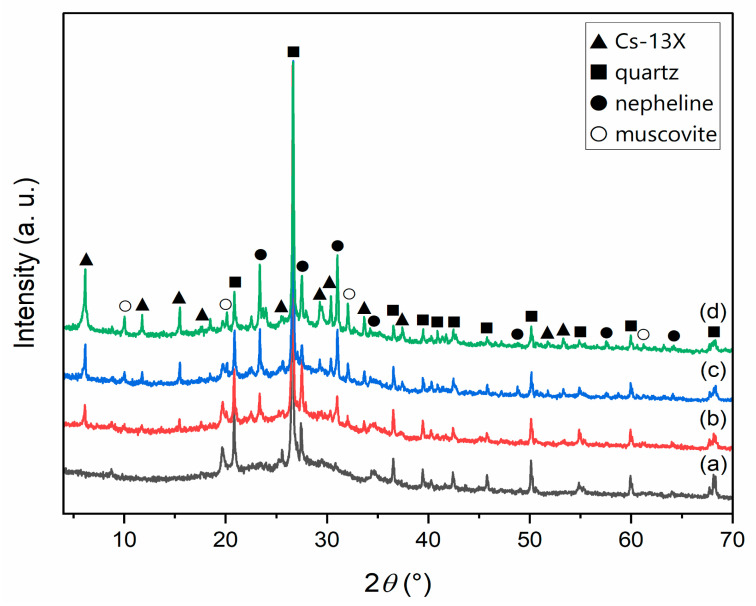
XRD patterns of geopolymer/Cs-13X zeolite composites with (**a**) 0 wt%, (**b**) 10 wt%, (**c**) 30 wt%, and (**d**) 50 wt% Cs-13X zeolite.

Unlike the ion-exchanged zeolite, which predominantly comprises a crystalline phase, the alkali-activated sample (geopolymer) predominantly comprises an amorphous phase, as confirmed by the characteristic amorphous “hump” observed in the 2*θ* range of 20–40° in the XRD pattern presented in Figure 4a. The amorphous phase is accompanied with the residual mineral phases that did not fully decompose during the alkali activation process. Quartz (PDF 01-087-2029) is identified as the dominant crystalline phase, while the other detected crystalline phases include muscovite (PDF 00-001-1098), along with weak reflections corresponding to the nepheline phase (PDF 00-19-1179). Mixing geopolymer precursors with Cs-13X zeolite leads to the formation of a geopolymer/Cs-13X zeolite composites during polymerization. The intensity of zeolite reflections increases with the amount of Cs-13X zeolite incorporated into the geopolymer matrix (Figure 4b–d). The most pronounced and intense zeolite-related peaks are observed in the sample containing 50 wt% Cs-13X zeolite (Figure 4d), indicating a higher proportion of preserved crystalline zeolite phase within the composite. In addition to the zeolite, all samples contain stable geopolymer phases, such as quartz, muscovite, and small amounts of nepheline (NaAlSiO_4_). It is evident that zeolite is less prone to geopolymerization than kaolin, leaving a considerable portion of the crystalline phase unreacted.

The obtained phase compositions can be explained by the fact that the high pH of the activating solution causes partial dissolution of the zeolite surface, leading to the formation of an interfacial transition zone enriched in aluminum relative to silicon. During this process, aluminates and silicates are released from the zeolite structure and subsequently participate in the geopolymerization reaction, contributing to the formation of the amorphous geopolymer network [28]. At the same time, zeolite dissolution leads to the release of Cs^+^ ions, which interact with the geopolymer matrix. It is assumed that ion exchange occurs between Cs^+^ and Na^+^ ions within the geopolymer, thereby effectively immobilizing cesium within the composite structure. This behavior indicates that the geopolymer matrix not only physically incorporates the zeolite but also chemically participates in the stabilization and immobilization of Cs ions.

**Figure 5:**
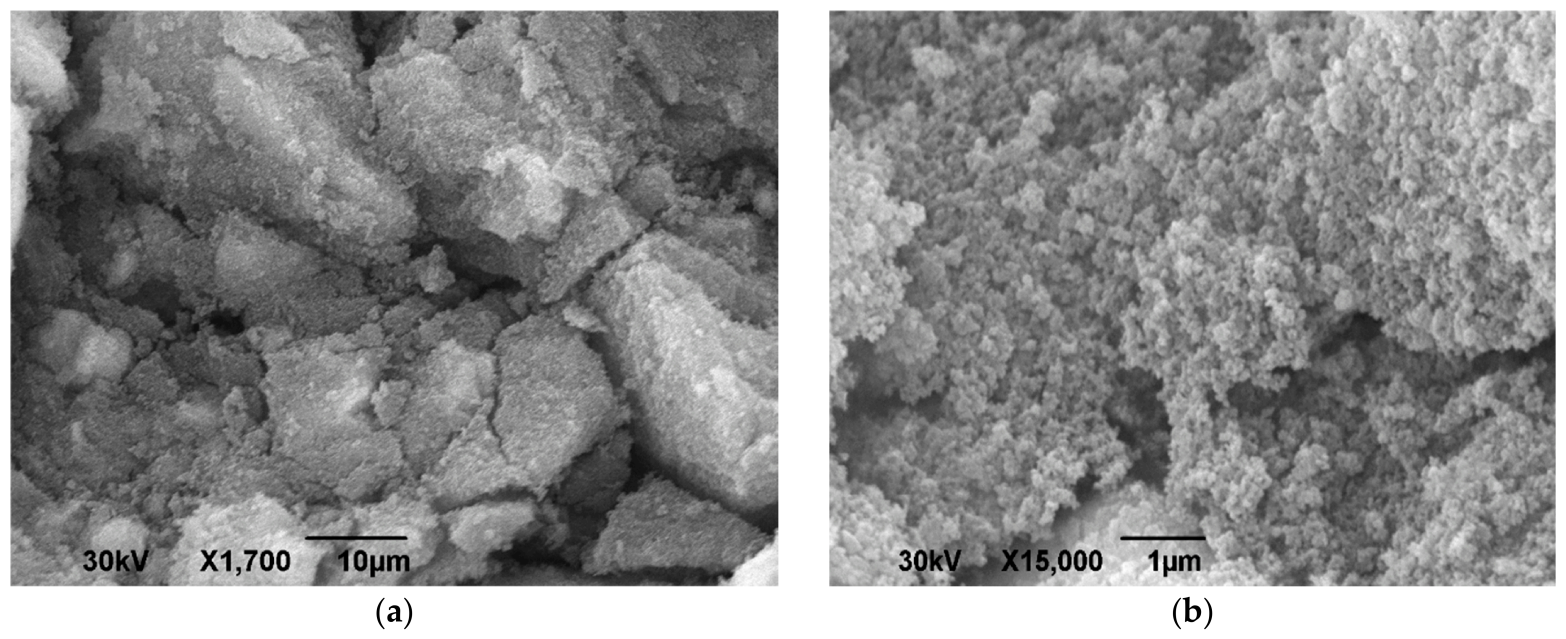
Fracture surface of metakaolin-based geopolymer at different magnifications: (**a**) 1700× and (**b**) 15,000×.

The SEM micrographs of the fracture surfaces of metakaolin-based geopolymers are presented in Figure 5. The micrograph obtained at the lower magnification (Figure 5a) reveals that the internal structure of the monolith consists of agglomerates with sizes of approximately 10 μm and interconnected pores several micrometers in diameter. The micrograph taken at the higher magnification (Figure 5b) shows the spherical morphology of the primary geopolymer particles that form the aforementioned agglomerates. In addition, pores and voids larger than 1 μm are clearly visible, confirming the presence of a porous network and indicating that the geopolymer monolith is a microporous material.

**Figure 6:**
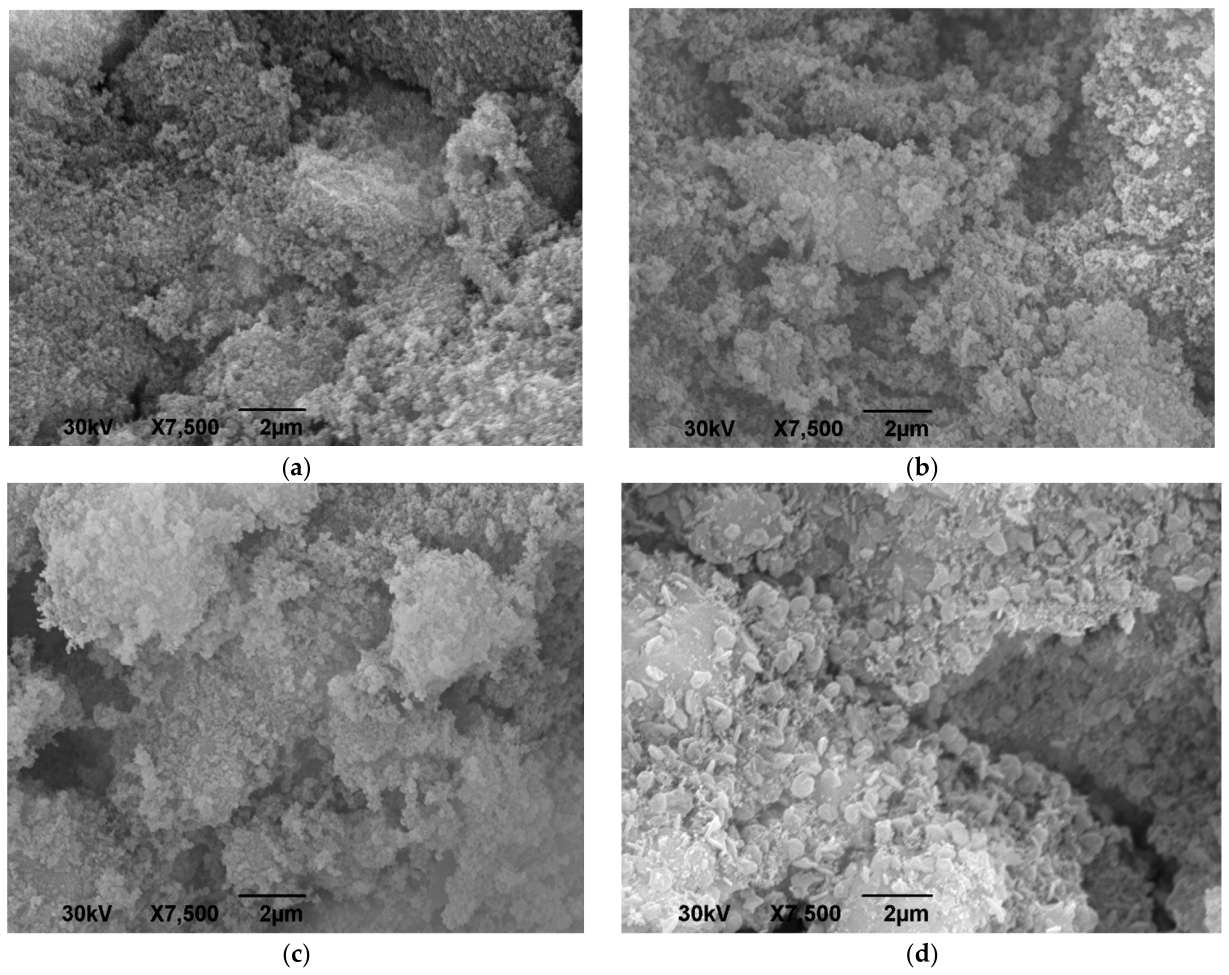
SEM micrographs of the fracture surfaces of geopolymer/Cs-13X zeolite composites containing (**a**) 0 wt%, (**b**) 10 wt%, (**c**) 30 wt%, and (**d**) 50 wt% Cs-13X zeolite.

SEM micrographs of the fracture surfaces of geopolymer/Cs-13X zeolite composites containing different amount of Cs-13X zeolite are presented in Figure 6. The microstructures of the composites containing 10 wt% (Figure 6b) and 30 wt% Cs-13X zeolite (Figure 6c) do not exhibit significant differences, either between each other or in comparison with the pure geopolymer (Figure 6a). The fracture surfaces of these samples are characterized by the presence of agglomerates and interconnected pores with sizes ranging from 2 to 10 μm. In addition, the spherical geopolymer particles, which constitute the building units of the agglomerated structure, are clearly visible on the fracture surfaces. On the other hand, the sample containing 50 wt% Cs-13X zeolite (Figure 6d) exhibits a markedly different morphology compared to the previously analyzed samples. The SEM micrograph reveals agglomerates approximately 10 μm in size, together with rhombohedral particles smaller than 0.5 μm. These particles are located on the surfaces of the geopolymer agglomerates or are partially embedded within the geopolymer matrix, indicating the presence and preservation of the crystalline zeolite phase within the composite. The obtained results are in excellent agreement with the XRD analysis results (Figure 4), indicating that characteristic zeolite peaks are the most pronounced and intense in the sample containing 50 wt% Cs-13X zeolite. This behavior confirms the increased fraction of the preserved crystalline zeolite phase in the composite structure at higher zeolite loadings. Furthermore, the fracture surface analysis indicates a predominantly brittle fracture behavior for all investigated samples.

**Figure 7:**
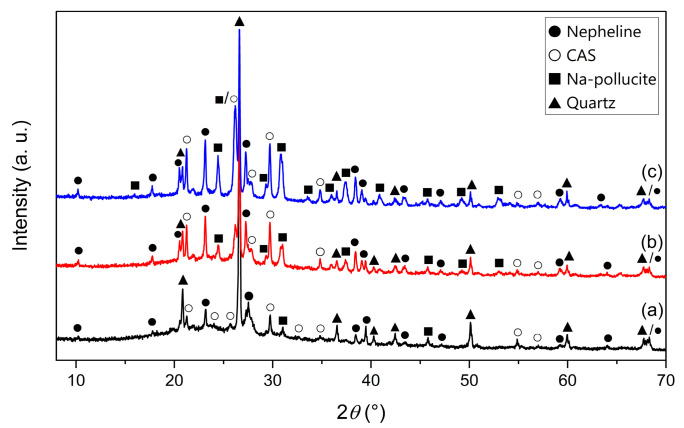
XRD patterns of the geopolymer/Cs-13X zeolite composites containing (**a**) 10 wt%, (**b**) 30 wt%, and (**c**) 50 wt% Cs-13X zeolite heat-treated at 950 °C (JCPDS; quartz: PDF- 00-046-1045, nepheline: 00-019-1176, Na-pollucite: 00-015-0317, CAS: 00-029-0406).

As previously stated in the Introduction, the obtained composite materials were heat-treated at 950 °C in order to remove all residual water from the samples. The temperature of heat treatment was relatively low in order to prevent potential evaporation of Cs. The XRD patterns of the composite samples heat-treated at 950 °C are presented in Figure 7. In addition to quartz, which remains stable and does not react with the other components, the formation of a nepheline phase and two stable Cs-containing aluminosilicate phases, pollucite (CsAlSi_2_O_6_) and CAS (cesium aluminosilicate, CsAlSi_5_O_12_), is observed. Nepheline (NaAlSiO_4_), which commonly forms in Na-rich geopolymer systems at elevated temperatures, is often considered as an intermediate or competing phase during the development of pollucite structures. Pollucite is formed through the thermally induced phase transformation of Cs-13X zeolite. For this reason, the amount of pollucite increases with the content of Cs-13X. The resulting pollucite is sodium-containing, which is expected considering that all samples contain significant amounts of sodium originating from both the geopolymer matrix and the zeolite itself. It is well-known that, under conventional ion-exchange conditions, complete replacement of Na^+^ ions by Cs^+^ ions in zeolite structures cannot be achieved, resulting in the retention of a certain amount of Na^+^.

In addition to Na-pollucite, a CAS phase is also formed. This compound is likewise a thermodynamically stable cesium aluminosilicate. As previously discussed, the highly alkaline activating solution causes partial dissolution of the zeolite surface. This dissolution results in the release of Cs^+^ ions, which subsequently interact with the geopolymer matrix. It is assumed that ion exchange occurs between Cs^+^ and Na^+^ ions within the geopolymer network, thereby promoting the effective immobilization of cesium in the composite structure. Upon heat treatment at 950 °C, the geopolymer material, comprising primarily metakaolin and sodium silicate, incorporates the released cesium and undergoes phase transformations, leading to the formation of the CAS phase. Therefore, the presence of the CAS phase provides further evidence that cesium released from zeolite during geopolymerization becomes incorporated into the geopolymer matrix and remains immobilized after thermal treatment. In addition to the crystalline phases, all samples contain a certain fraction of an amorphous phase.

It is crucial to point out that, during heat treatment, the system undergoes a series of physical and chemical transformations that convert the initial amorphous structure into thermodynamically stable crystalline phases [36,44,45]. The first stage involves dehydration, during which free, zeolitic, and chemically bound water is progressively removed. The water loss causes shrinkage of the geopolymer network, increases material density, and enhances the mobility of Na^+^ and Cs^+^ ions, creating favorable conditions for structural rearrangement. As the temperature increases, the geopolymer and zeolite frameworks progressively collapse. Above approximately 700–800 °C, the amorphous aluminosilicate network becomes thermodynamically unstable, accompanied by the partial disruption of Si–O–Al bonds and enhanced alkali-ion diffusion, which promote the formation of locally ordered aluminosilicate domains that subsequently act as nucleation sites for crystallization. At 950 °C, recrystallization occurs, producing crystalline phases identified by X-ray diffraction (XRD). Nepheline (NaAlSiO_4_) forms as an expected product because it is one of the most stable sodium aluminosilicate phases in systems with relatively low Si/Al ratios [46]. Its formation indicates that sodium released from the geopolymer network, together with aluminum supplied by metakaolin, participates in crystallization under favorable local stoichiometric conditions. A particularly important result is the formation of Na-pollucite. Although the ideal composition of pollucite is CsAlSi_2_O_6_, geopolymer systems commonly form solid solutions of the type (Cs,Na)AlSi_2_O_6_ due to the partial substitution of Cs^+^ by Na^+^ [47,48]. This indicates that cesium diffuses from zeolite particles during sintering, mixes with sodium from the geopolymer matrix, and co-crystallizes into a stable aluminosilicate phase. The formation of Na-pollucite also suggests an excess of sodium, which contributes to stabilization of the crystal lattice. In addition to Na-pollucite, a cesium aluminosilicate (CAS) phase was detected, indicating that not all cesium was incorporated into the pollucite structure. This can be attributed to limited Cs^+^ diffusion during sintering and local compositional heterogeneities originating from zeolite particles, promoting the formation of multiple cesium-containing aluminosilicate phases. Overall, thermal treatment involves dehydration, framework collapse, enhanced alkali-ion mobility, and recrystallization, leading to the transformation of the amorphous geopolymer into stable crystalline phases. Sodium is primarily incorporated into nepheline and Na-pollucite, while cesium is distributed between Na-pollucite and the CAS phase depending on local composition and diffusion during heat treatment.

The CAS phase possesses a one-dimensional channel system comprising eight-membered rings extending along the *z*-axis. The effective pore dimensions of these channels are approximately 4.7 × 2.4 Å. In contrast, pollucite exhibits a three-dimensional channel system consisting of curved eight-membered rings with effective pore openings (free channel diameters) of approximately 4.2 × 1.6 Å [49]. Considering that the ionic diameter of Cs^+^ is approximately 3.34 Å, once the CAS phase or pollucite is formed, cesium ions become effectively trapped within the aluminosilicate framework. Consequently, their migration from the crystal structure into the surrounding environment is highly restricted, contributing to the long-term immobilization of cesium.

It is important to emphasize that the Na-pollucite phase obtained in this study was formed at a lower temperature than that typically required during the conventional heat treatment of Cs-exchanged zeolites. Under conventional processing conditions in air, the formation of Cs-containing aluminosilicate phases, including pollucite and the CAS phase, is generally reported at temperatures around 1100 °C [45,50]. In the present work, the Na-pollucite phase was successfully formed at temperatures as low as 950 °C. This temperature difference should be considered in the context of Na incorporation since Na-pollucite is known to crystallize at lower temperatures than pure Cs-pollucite. Nevertheless, the formation of the desired Cs-containing aluminosilicate phase at 950 °C represents a reduction in the thermal treatment temperature compared with conventional processing of Cs-exchanged zeolites, and is comparable to the temperatures reported for Cs-containing aluminosilicate phase formation during hot-pressing processes [51,52]. The lower processing temperature can contribute to reduced energy consumption and may help minimize cesium volatilization while still ensuring effective removal of physically and chemically bound water from the material.

The crystallinity of samples heated at 950 °C was determined and is presented in Table 4. The geopolymer sample containing 10 wt% Cs-13X zeolite exhibited a higher proportion of the amorphous phase at 950 °C, with an overall crystallinity of only 41%. As expected, the fraction of the amorphous phase decreased with increasing Cs-13X content, while the fraction of the crystalline phase increased. Accordingly, a higher percentage of Cs-13X zeolite led to an increase in the crystalline phase content. The crystallinity of sample containing 50 wt% Cs-13X reached value of 72%.

The phase composition of geopolymer/Cs-13X zeolite composites with different amounts of Cs-13X zeolite heat-treated at 950 °C is presented in Table 5. Based on the obtained data on the total percentage of all phases in individual samples, it can be seen that in the geopolymer/Cs-13X zeolite composite with 10 wt% Cs-13X zeolite after heat treatment at 950 °C, a dominant amorphous structure is present with a fraction of 59%, indicating that the largest part of the material remained in a disordered, glassy state. In addition to the amorphous phase, several crystalline phases were identified, indicating partial crystallization of the system. The most abundant crystalline phase is quartz (22.3%), confirming the presence of a stable SiO_2_ component in the sample. The presence of nepheline (9.8%) was also recorded. Cs-aluminosilicate phases are present in a very low percentage (approximately 9%), which was expected since only 10 wt% of zeolite was added compared to 90 wt% kaolin. In this sample, the CAS phase is present in a higher percentage than pollucite. The CAS phase is present at 7.9%, while the Na-pollucite phase is present at about 1%, indicating that in this case the dissolution process of the zeolite surface is dominant due to high pH, and that Cs is incorporated into the geopolymer matrix prior to firing. Although the obtained results indicate that the thermal treatment of the geopolymer/Cs-13X zeolite composite with 10 wt% Cs-13X at 950 °C leads to the development of several crystalline phases, the amorphous matrix still remains the dominant component of the system. The heat-treated sample with 30 wt% Cs-13X zeolite also contains a significant fraction of the amorphous phase (42%), indicating that a large portion of the material remains in a disordered, amorphous state even after heating at 950 °C (Table 5). Among the crystalline phases, quartz is the most abundant (21.5%), confirming the stability of the silica (SiO_2_) structure in the sample with 30 wt% Cs-13X zeolite. Nepheline is slightly more abundant (10.3%) when compared to the composite sample with 10 wt% Cs-13X sample, due to the higher zeolite content in the system. The significant portion of Cs aluminosilicate phases is also present in sample with 30 wt% Cs-13X. To be more precise, approximately 15% CAS phase and over 11% Na-pollucite are present in the composite sample with 30 wt% Cs-13X (Table 5). These two phases contribute to the stable binding and immobilization of cesium within the crystalline structure. Overall, the system shows a balance between the amorphous matrix and developed crystalline phases, whereas thermal treatment at 950 °C leads to pronounced crystallization without complete loss of the amorphous structure.

For the geopolymer/Cs-13X zeolite composite with 50 wt% Cs-13X heat-treated at 950 °C, the dominant cesium-related phases are the CAS phase (22.3%) and Na-pollucite (27.1%). This means that almost half of the total material (~49%) is present in crystalline Cs-bearing phases, indicating very efficient incorporation of cesium into the aluminosilicate matrix. The amorphous phase (28%) shows that part of the geopolymer gel did not crystallize even after treatment at 950 °C. However, the reduction in amorphous content indicates progressive crystallization and structural reorganization during heating due to the higher proportion of added Cs-13X zeolite. The presence of quartz (9.3%) likely originates from residual unreacted silicate components and represents a thermally stable inert phase. Its relatively low content suggests that a significant portion of Si participated in the formation of new crystalline aluminosilicates. The presence of nepheline (13.3%) confirms intensive crystallization of Na-aluminosilicate structures during the heat treatment. It often forms in Na-rich geopolymer systems at high temperatures and represents either an intermediate phase or a competing phase during the development of pollucite structures. It is very difficult to compare the phase composition of the materials obtained in this study with those reported in other publications because neither identical starting materials nor the same processing conditions were used. In a study by Li et al. [53], KOH was used as the alkaline activator, resulting in the formation of leucite instead of nepheline. This clearly demonstrates that even minor changes in the synthesis procedure can significantly affect the final phase composition of the material.

**Figure 8:**
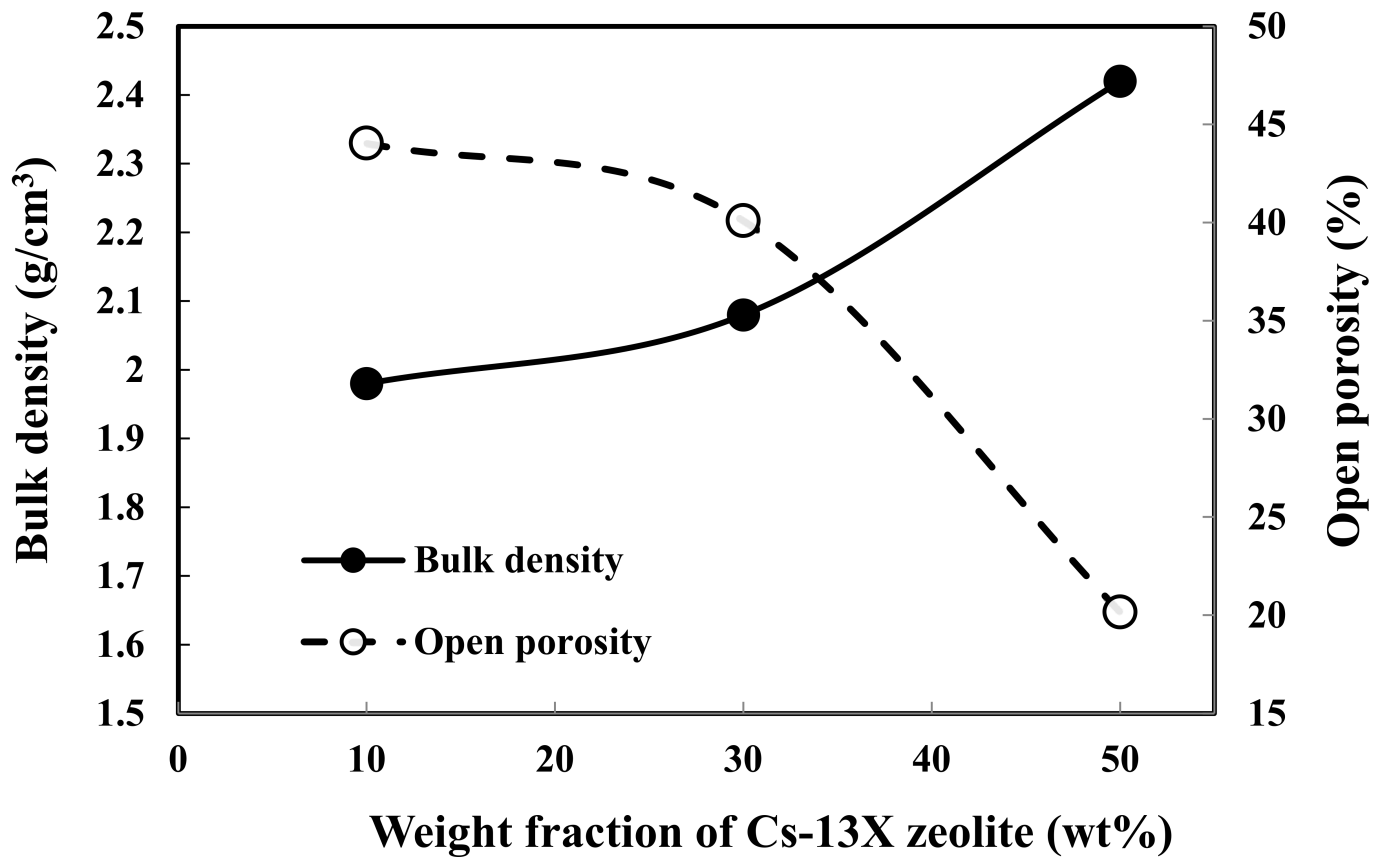
Density and open porosity of geopolymer/Cs-13X zeolite composites heat-treated at 950 °C for 2 h containing different amounts of Cs-13X zeolite.

The effects of zeolite content on the open porosity and density of heat-treated geopolymer/Cs-13X zeolite composites are presented in Figure 8. As can be seen, the density increases with Cs-13X zeolite content, whereas open porosity decreases, indicating fast densification of zeolite. The sample with 10 wt% zeolite exhibits the lowest density of 1.98 g/cm^3^ and, consequently, the highest open porosity. A slightly higher density and approximately for 4% lower porosity are observed for the sample containing 30 wt% zeolite. In both samples, the amorphous disordered phase is dominant, while quartz represents the main crystalline phase. The highest density (2.42 g/cm^3^) and the lowest open porosity (only ~20%) are observed in the sample with the highest zeolite content (50 wt%), in which Cs-aluminosilicate phases dominate (~49%). The reduction in the amorphous phase content, as previously noted, indicates more intensive crystallization and phase transformation during heating due to the higher amount of Cs-13X zeolite. During crystallization, the particles arrange themselves into a more ordered crystal lattice, which usually allows for denser packing and a reduction in volume for the same mass. Such structural changes lead to a significant decrease in open porosity and a simultaneous increase in material density.

**Figure 9:**
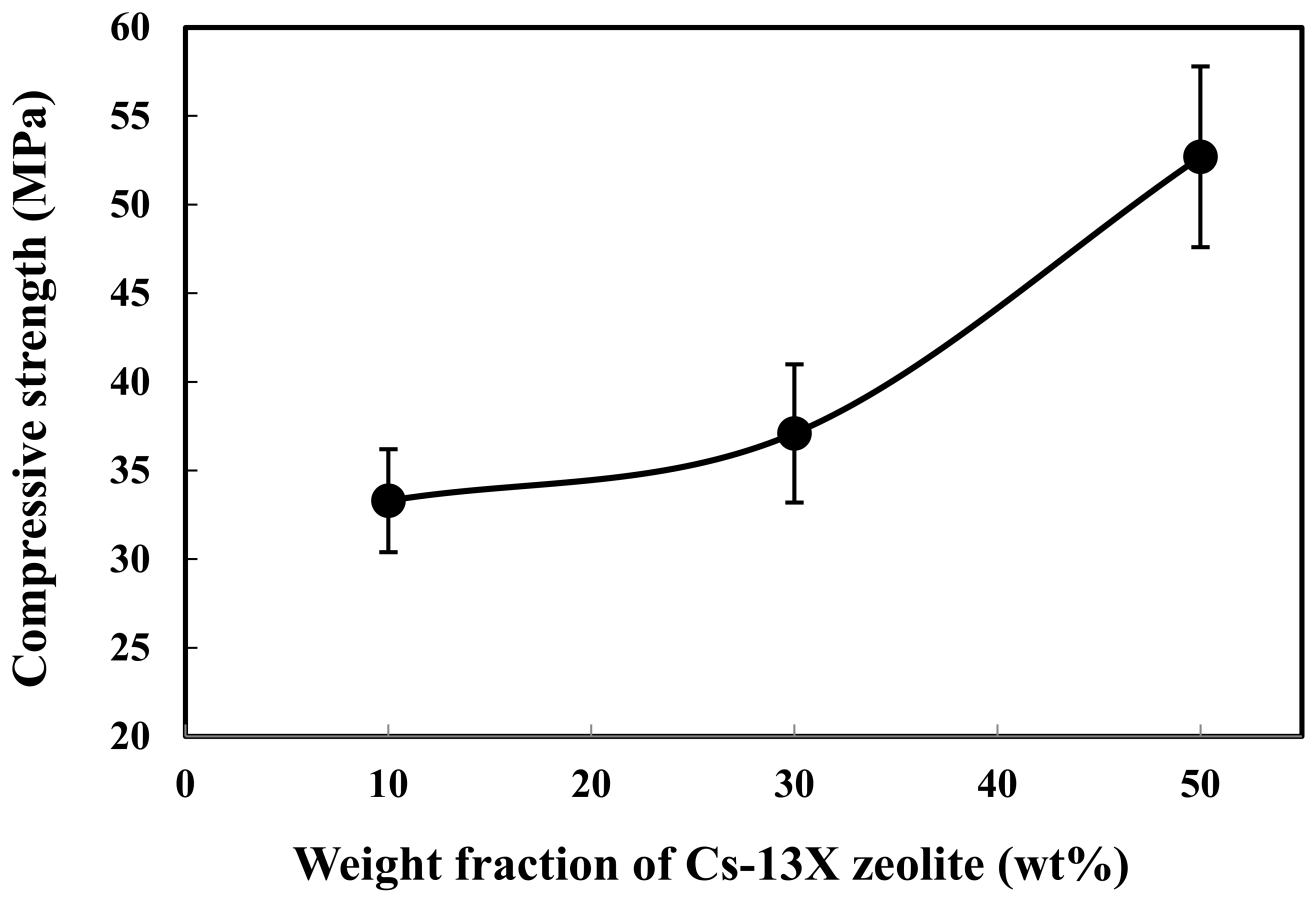
Compressive strength of geopolymer/Cs-13X zeolite composites heat-treated at 950 °C for 2 h containing different amounts of Cs-13X zeolite.

One of the most important properties of geopolymer–zeolite composites materials is compressive strength, as sufficiently high values are essential for the safe handling and disposal of radioactive waste. The mean compressive strength values of the samples are presented in Figure 9. Similar to density, the compressive strength of geopolymer–zeolite composites increases with the Cs-13X zeolite content. This is expected given that compressive strength is proportional to density and inversely proportional to porosity. The highest compressive strength (~53 MPa) was measured for samples containing 50 wt% Cs-13X zeolite, resulting from the highest content of crystalline phase (72%, Table 4.) and the lowest porosity value (Figure 8). Therefore, it can be concluded that both porosity and the amount of crystalline phases in the samples strongly influence the compressive strength. The highly ordered crystalline structure is expected to have higher compressive strength than the amorphous phase. The compressive strength of samples containing 30 wt% zeolite was 37.1 MPa, whereas the lowest compressive strength of 33.3 MPa was observed for samples containing 10 wt% zeolite. This finding is also consistent with their structural characteristics, as these samples exhibit relatively high porosity and a lower content of crystalline phases, particularly Cs-aluminosilicate phases. Similar to the phase composition, a direct comparison of the compressive strength values obtained in this study with those reported in the literature is challenging. Even minor variations in chemical composition, starting materials, or experimental procedures can significantly affect the compressive strength of geopolymer/zeolite composites. For example, Xu et al. [29] reported a compressive strength of 26.7 MPa for a geopolymer/zeolite composite heat-treated at 1000 °C, whereas Li et al. [53] reported a value of 84.5 MPa for a geopolymer/zeolite composite heat-treated at 1100 °C. Nevertheless, the most important consideration is that the compressive strength is sufficient to ensure safe and easy handling of the samples, which can be concluded for the measured value of ~33 MPa.

**Figure 10:**
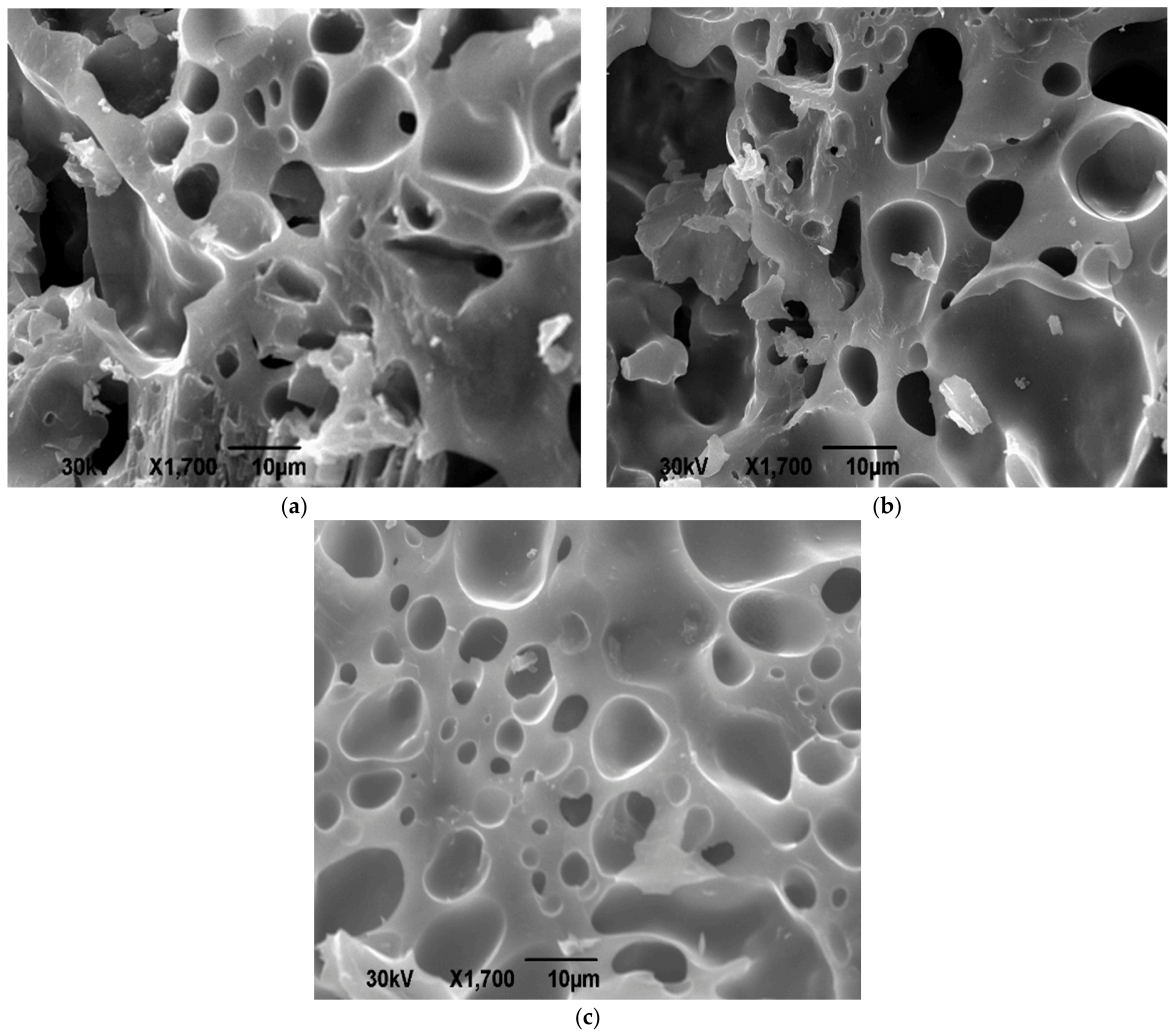
SEM micrograph of geopolymer/Cs-13X zeolite composite samples heat-treated at 950 °C for 2h containing (**a**) 10 wt%, (**b**) 30 wt%, and (**c**) 50 wt% of Cs-13X zeolite.

Figure 10 shows the SEM micrographs of composite samples heat-treated at 950 °C. All of the samples have been imaged at a fracture section of the geopolymer-based ceramics. Heat treatment at 950 °C leads to significant morphological changes in the geopolymer–zeolite composites. The initially porous geopolymer gel network undergoes sintering (densification) process resulting in a smoother, more continuous, and denser fracture surface. During the thermal treatment, the geopolymer–zeolite composites partially melt and subsequently solidify, leading to the formation of pores of various sizes throughout the material. The SEM micrographs confirm the porosity characteristics discussed previously. Fracture surface images of the samples containing 10 wt% and 30 wt% Cs-13X zeolite reveal a highly porous microstructure. These samples are characterized predominantly by open porosity, whereas closed porosity is present to a much lesser extent. The pores exhibit irregular shapes and a heterogeneous size distribution, with diameters ranging from approximately 1 μm to more than 20 μm. The presence of large, interconnected pores suggests incomplete densification during the sintering process. In contrast, the sample containing 50 wt% Cs-13X zeolite exhibits a distinctly different microstructure. Both open and closed porosity are present; however, the pores are considerably smaller and more uniformly distributed throughout the matrix. The size of the closed pores generally does not exceed 20 μm, indicating a higher degree of densification and more effective sintering. Furthermore, this sample contains the lowest content of amorphous phase compared with the composite samples containing 10 wt% and 30 wt% Cs-13X zeolite. The reduced amorphous content was followed by more extensive crystallization and more efficient particle bonding during thermal treatment. As a result, a denser and more homogeneous microstructure was formed, characterized by a smaller number of large pores and structural defects.

## 4. Conclusions

An efficient method for the removal of Cs ions from aqueous solution and their containment was presented. Cs ions were removed from CsCl water solution by ion exchange using Na-13X zeolite. Approximately two-thirds of the Na^+^ cations in starting Na-13X zeolite sites were replaced with Cs^+^ during ion exchange. The obtained Cs-13X zeolite was subsequently combined with kaolinite to prepare geopolymer/Cs-13X zeolite composites containing 10–50 wt% Cs-13X zeolite. During geopolymerization, a smaller portion of Cs^+^ was released from the zeolite and incorporated into the geopolymer matrix, while the remaining Cs remained immobilized within the zeolite structure. Heat treatment of the geopolymer/Cs-13X zeolite composites at 950 °C induced the crystallization of two stable Cs-containing phases. The Cs incorporated into the geopolymer matrix crystallized as the CAS phase, whereas the Cs retained in the Cs-13X zeolite crystallized as pollucite. The crystallinity degree increased with the amount of Cs-13X zeolite, reaching value of 72% in samples containing 50 wt% Cs-13X zeolite. Similar to the crystallinity, the density and compressive strength of the heat-treated composites increased with increasing Cs-13X zeolite content due to the crystallization of the amorphous phase. The highest compressive strength, approximately 53 MPa, was achieved for the heat-treated composites containing 50 wt% Cs-13X zeolite. This level of mechanical strength is sufficient to ensure safe handling and long-term containment of the immobilized Cs ions.

The proposed method offers several advantages for the treatment and immobilization of radioactive cesium-containing waste. Incorporation of Cs-loaded zeolite into a geopolymer matrix provides dual immobilization, with Cs retained in both the zeolite structure and the newly formed crystalline phases after heat treatment. Thermal treatment at 950 °C promotes the formation of two stable Cs-bearing phases, pollucite and CAS, enhancing the long-term durability of the waste form. The use of inexpensive and widely available raw materials, such as kaolinite and zeolite, also supports the economic feasibility of large-scale application. Further studies are needed to assess the process using real radioactive waste streams with more complex chemical compositions. Overall, the combined ion-exchange–geopolymer approach shows strong potential for safe, long-term cesium immobilization, although further optimization is required to improve energy efficiency and confirm large-scale performance.

## Figures and Tables

**Table 1:** Chemical composition of metakaolin (MK).

Oxide (wt%)	Na_2_O	MgO	Al_2_O_3_	SiO_2_	P_2_O_5_	K_2_O	CaO	TiO_2_	MnO	Fe_2_O_3_	ZnO	As_2_O_3_	BaO	LOI * 950 °C
MK	0.17	0.53	31.23	60.85	0.03	2.29	0.40	0.65	0.01	1.92	0.01	0.08	0.05	1.61

* Lost of ignition.

**Table 2:** Weight fractions of metakaolin (MK) and Cs-13X zeolite in solid-phase precursors for alkali-activated geopolymer/Cs-13X zeolite composites.

MK (wt%)	Cs-13X (wt%)
100	/
90	10
70	30
50	50

**Table 3:** XRF analysis of the composition of Na-13X zeolite before and after Cs^+^ ion exchange.

Elements (wt%)	Na	Al	Si	O	Cs	Brutto Formula
Before exchange	15.10 ± 0.14	18.56 ± 0.08	21.75 ± 0.11	43.87 ± 0.21	0.00 ± 0.00	Na_0.96_Al_1.00_Si_1.13_O_4_
After exchange	5.28 ± 0.09	14.81 ± 0.11	18.89 ± 0.14	40.61 ± 0.24	20.42 ± 0.24	Na_0.36_Cs_0.24_Al_0.87_Si_1.06_O_4_

**Table 4:** The crystallinity of the geopolymer/Cs-13X zeolite composite samples heat-treated at 950 °C for 2 h containing different amounts of Cs-13X zeolite.

Weight Fraction of Cs-13X Zeolite	Crystallinity
10%	41%
30%	58%
50%	72%

**Table 5:** Phase composition of geopolymer/Cs-13X zeolite composites heat-treated at 950 °C with different weight fractions of Cs-13X zeolite. The estimated standard deviations are given in round brackets.

Identified Phases (%)	Weight Fraction of Cs-13X Zeolite		
	10 wt%	30 wt%	50 wt%
Amorphous	59.0 (19)	42.0 (18)	28.0 (16)
Quartz	22.3 (8)	21.5 (16)	9.3 (3)
Nepheline	9.8 (8)	10.3 (4)	13.3 (3)
CAS	7.9 (8)	15.0 (15)	22.3 (7)
Na-pollucite	>1.0 (2)	11.2 (3)	27.1 (7)

## Data Availability

The original contributions presented in this study are included in the article. Further inquiries can be directed to the corresponding author.
